# Gender and Spondyloarthropathy-Associated Uveitis

**DOI:** 10.1155/2013/928264

**Published:** 2013-12-22

**Authors:** Wendy M. Smith

**Affiliations:** Mayo Clinic, 200 First Street SW, Rochester, MN 55905, USA

## Abstract

Spondyloarthropathies encompass a group of inflammatory diseases with arthritis and other features such as enthesitis and dermatologic and gastrointestinal involvement. Up to 37% of spondyloarthropathy patients may develop uveitis which is typically bilateral asynchronous acute anterior uveitis. Spondyloarthropathies with and without uveitis are more prevalent among males; the reasons for gender imbalance are unclear. This review will focus on gender differences in the prevalence, incidence, clinical manifestations, and prognosis of uveitis associated with spondyloarthropathies.

## 1. Introduction

Spondyloarthropathies are a group of inflammatory diseases with overlapping features including arthritis of the axial skeleton and/or peripheral joints, inflammatory back pain, enthesitis, and dermatologic and gastrointestinal involvement. The majority of these syndromes are more prevalent in males although the reasons for this gender imbalance are unclear. The concept of individualized medicine has become increasingly important; therefore, a better understanding of gender differences in uveitis may help us provide better care and prevent vision loss. This review will focus on gender differences in the prevalence, incidence, clinical manifestations, and prognosis of uveitis in spondyloarthropathies.

Most spondyloarthropathy patients are positive for the human leukocyte antigen (HLA)-B27 allele, although the prevalence varies: 90% ankylosing spondylitis, 40–80% reactive arthritis, 40–50% psoriatic arthritis, 35–75% enteropathic arthritis, and 70% undifferentiated spondyloarthropathy [1, 2]. Uveitis may occur in up to 37% of spondyloarthropathy patients [1, 2] and typically manifests as bilateral asynchronous acute anterior uveitis although posterior segment manifestations including vitritis, papillitis, and cystoid macular edema may occur in 20–30% of cases [3, 4]. HLA-B27-associated acute anterior uveitis (AAU) is one of the most common types of uveitis in the United States and other Western countries, accounting for approximately 30% of uveitis cases and 50–80% of anterior uveitis [2, 5–8]. Since most patients with spondyloarthropathies and uveitis are HLA-B27 positive, studies assessing the demographics, clinical course, management, complication rates, and prognosis generally make comparisons to HLA-B27-negative uveitis patients.

## 2. Gender Differences in Prevalence and Incidence

In general, multiple studies have shown that males are more likely to develop HLA-B27-associated AAU than females with male-to-female ratios ranging from 1.1 to 2.5 to 1 [2, 3, 9–17]. A few studies have reported higher ratios (i.e., 3.5 to 1 by Wakefield et al.) [9] Table 1 summarizes the studies reviewed in this paper.

## 3. Gender Differences in Clinical Manifestations

Many, but not all, patients with HLA-B27-associated AAU have spondyloarthropathy at the time of uveitis diagnosis; systemic inflammatory disease can also develop after the onset of uveitis. Men are more likely to develop ankylosing spondylitis and commonly have a more severe course of disease in terms of radiographic changes [18]. Women usually have a milder course of systemic disease with less typical features such as peripheral arthritis [2].

Wakefield et al. [9] identified 41 consecutive patients with HLA-B27 and anterior uveitis at an Australian clinic; some also had posterior uveitis, panuveitis, or retinal vasculitis. The HLA-B27-positive males were more likely to have spondyloarthropathy than the B27-positive females and the B27-negative patients.

In a study from The Netherlands, Linssen and Meenken [11] performed a prospective study comparing 119 patients with HLA-B27-positive AAU to 35 B27-negative cases. The HLA-B27-positive AAU patients were more likely to be male. Initially, about half of the HLA-B27-positive patients also had spondyloarthropathy; after nine years, two-thirds had been diagnosed with spondyloarthropathy. Among the females without ankylosing spondylitis, there was a nonstatistically significant trend towards older age at onset of AAU.

A total of 148 patients with HLA-B27-associated uveitis from two uveitis practices in Ohio and Maryland were assessed in a retrospective study by Tay-Kearney et al. [12] There was no gender difference in the onset of uveitis (acute versus insidious) or the presence of hypopyon, increased intraocular pressure, posterior synechiae, cataract, or cystoid macular edema. There was also no association between gender and the presence of systemic disease although women seemed more likely to have atypical spondyloarthropathies such as incomplete Reiters syndrome or undifferentiated spondyloarthropathy.

Lyons and Rosenbaum [19] reported the characteristics of eye disease in 17 patients with uveitis and inflammatory bowel disease (IBD) from a university clinic in Oregon. In comparison to 89 uveitis patients with spondyloarthropathy, uveitis associated with IBD was more likely to occur in females 82% (*P* < 0.001) and less likely to be associated with HLA-B27 (*P* < 0.01). The inflammation was also more likely to be insidious rather than sudden onset (*P* < 0.001) and chronic (*P* < 0.001). Uveitis with IBD was often bilateral and simultaneous with a significant posterior component. Cataract and glaucoma were more commonly associated with IBD and uveitis versus spondyloarthropathy-related uveitis.

In another study from the Oregon group, Paiva et al. [20] assessed 16 patients with uveitis and psoriatic arthritis in comparison to 89 uveitis patients with spondyloarthropathy. The psoriatic arthritis patients with uveitis and only axial joint involvement were more likely to be male (8 of 8) and HLA-B27 positive (6 of 6 tested) compared to those with uveitis and peripheral arthritis only. The difference between the cohorts was very small.

Queiro et al. [21] conducted a retrospective study of 71 psoriatic arthritis patients in Spain and found 13 (18%) with uveitis. Among the small number of patients with uveitis, there was a slight predominance of females. Interestingly, multivariate analysis showed a significant association between psoriatic arthritis patients with uveitis and HLA-DR13 (*P* = 0.0056), bilateral sacroiliitis, and syndesmophytes. HLA-B27 was associated in a univariate analysis (*P* = 0.026) but did not reach statistical significance in a logistic regression model. The number of uveitis cases was very small in this study as well.

In an observational case series from France, Monnet et al. [13] studied the ocular and extraocular manifestations in 99 patients with HLA-B27-associated uveitis. Although the male-to-female ratio in the group of patients with spondyloarthropathy and uveitis was higher than in those without spondyloarthropathy, the difference was not statistically significant in a Chi square test (*P* = 0.06).

Braakenburg et al. [14] investigated gender differences in HLA-B27-associated AAU through a retrospective study of 177 patients with at least one year of followup at a university practice in The Netherlands. The male-to-female ratio was 1.1 : 1. There was no difference between males and females in terms of average age at onset of uveitis; however, the onset of systemic HLA-B27-associated systemic disease occurred earlier in men than in women (30 versus 37 years; *P* = 0.021) and men were more likely to develop ankylosing spondylitis. Men were also more likely to develop systemic disease prior to the onset of uveitis whereas systemic disease in women was often diagnosed after uveitis. Over time, the gender differences in risk of systemic HLA-B27-associated disease diminished as more patients were diagnosed.

A retrospective study of 504 Chinese/Taiwanese patients with HLA-B27 uveitis was conducted by Chung et al. with longitudinal followup over 18 years [15]. In this cohort, males were significantly older than females at the onset of uveitis (*P* = 0.004). 76.8% of patients had spondyloarthropathy; among these patients, ankylosing spondylitis occurred more frequently in males (50.1% versus 27.2%, *P* < 0.001) whereas undifferentiated spondyloarthropathy was more common in females (40.8% versus 24.2%, *P* < 0.001). Uveitis without spondyloarthropathy showed no predilection for gender.

In a California study, Loh and Acharya [17] found that HLA-B27-associated systemic diseases were slightly more common in men (46%) than in women (41%) with acute and chronic HLA-B27-associated uveitis although the difference was not statistically significant. Finally, Zheng et al. [22] studied 240 Chinese patients with HLA-B27-associated AAU. The mean age of onset was younger in the male patients (36.0 versus 39.5, *P* < 0.05). Male patients were also more likely to have concomitant spondyloarthropathy, especially ankylosing spondylitis.

In summary, HLA-B27-associated uveitis patients are more likely to be male and to have systemic inflammatory disease, in particular ankylosing spondylitis. Male patients also tend to be younger at the onset of uveitis. Women might be more prone to atypical or undifferentiated spondyloarthropathies. These gender differences may lessen as time elapses from initial uveitis presentation, since male patients seem to be more likely to have systemic disease prior to the onset of uveitis, but, as more female patients develop systemic manifestations after uveitis onset, the ratio becomes nearly equal. Small studies of uveitis patients with psoriatic arthritis and IBD show trends towards a female predominance, uveitis onset more insidious than typical HLA-B27-associated AAU, less association with HLA-B27, and atypical spondyloarthropathy. The different and sometimes conflicting conclusions may be due to variable cohort sizes, different methods of diagnosing spondyloarthropathies, and the inclusion or exclusion of chronic uveitis.

## 4. Gender Differences in Treatment and Prognosis

Some studies have suggested that HLA-B27-associated uveitis has a poorer prognosis than HLA-B27-negative uveitis [3, 10]. Power et al. [3] studied 191 consecutive HLA-B27-positive uveitis patients with and without systemic inflammatory disease and compared them to 72 patients with idiopathic HLA-B27-negative uveitis. The HLA-B27-associated uveitis patients had significantly higher mean recurrence rates and more complications such as cystoid macular edema, extensive and persistent posterior synechiae, secondary glaucoma, vitritis, and papillitis. Not unexpectedly, these patients required more periocular and systemic corticosteroids as well as surgical interventions. Finally, there was a higher rate of legally blind eyes among the HLA-B27-positive uveitis cases. In contrast, other studies have shown a higher rate of complications in HLA-B27-negative uveitis plus more recalcitrant inflammation [11, 23]. Differences between the genders in terms of prognosis and treatment of HLA-B27-associated uveitis are even less well defined.

Wakefield et al. [9] found that uveitis in HLA-B27-positive males with spondyloarthropathy occurred at a younger age but with fewer recurrences compared to the B27-positive females without associated rheumatologic disease. The statistical significance of this data was not stated, and the number of patients in each group was small (HLA-B27 and associated rheumatologic disease: 22 males, 1 female. HLA-B27 and no associated rheumatologic disease: 10 males and 8 females).

Monnet et al. [13] observed no significant gender differences in rates of hypopyon, posterior synechiae, cataract, intraocular pressure, papillitis, cystoid macular edema, final visual acuity, or treatments. Family history of spondyloarthropathy with or without uveitis and extraocular symptoms were also similar between the genders. Uy et al. [24] also found no relationship between gender and the risk of cystoid macular edema.

Braakenburg et al. [14] determined that the average number of attacks of AAU per year was not affected by gender although women did require longer topical treatment of active episodes. Over time there was a nonsignificant trend towards more attacks in women. Bilateral involvement occurred more frequently in women, although there were no gender differences in the rates of developing chronic uveitis or attacks longer than six months. Clinical manifestations or complications of inflammation such as posterior synechia, anterior chamber fibrin, hypopyon, vitritis, and cystoid macular edema were equally likely in men and women. Occlusive retinal vasculitis did occur more frequently in men (8% versus zero, *P* = 0.39), three of whom had HLA-B27-associated systemic disease, but not inflammatory bowel disease or Behçet's disease. There was no difference in the rates of secondary glaucoma, cataract, vision loss, and surgical interventions. In terms of treatment, periocular steroids, systemic steroids, and nonsteroid systemic immunosuppressants were used equally in male and female patients.

Chung et al. [15] found no gender difference in the mean number of recurrent attacks of AAU in a cohort of Taiwanese patients; however, the frequency of attacks was significantly higher among women (80/100 patient-years versus 68, *P* = 0.002). In the first five years after the initial attack, there was no difference in the frequency of attacks. More than five years afterward, a higher rate of recurrence occurred in female patients (52/100 patient-years versus 39, *P* = 0.008).

In a retrospective study Agnani et al. [16] identified 207 patients with acute anterior uveitis who were HLA-B27 positive or who had a diagnosis with axial spondyloarthritis. The probability of recurrence within a year was similar between men and women by Kaplan-Meier estimates. Univariate analysis with a Cox proportional hazards model showed that male gender was significantly associated with a shorter time interval between attacks of recurrent anterior uveitis.

Loh and Acharya [17] performed a retrospective chart review of 99 HLA-B27-associated uveitis patients. They found no statistically significant gender difference in the rate of developing chronic uveitis versus recurrent disease. Male gender was a risk factor for vision loss (20/50 or worse on a single visit) in a backward stepwise multivariate regression.

In summary, although males are probably more likely to develop HLA-B27-associated uveitis at a younger age, females may have more frequent recurrences of inflammation and may require longer topical treatment. In contrast, some studies found that males might be more prone to recurrent attacks of inflammation. One study found that male gender is a risk factor for single visit vision loss of 20/50 or worse, and another found that males were more likely to have occlusive vasculitis. Otherwise, there appeared to be no gender differences in the risks of hypopyon, vitritis, papillitis, cataract, cystoid macular edema, intraocular pressure, secondary glaucoma, or final visual acuity. There were also no significant differences in types of treatments.

## 5. Possible Explanations for the Gender Differences

It is difficult to determine the precise mechanism(s) for these differences as few studies have been specifically designed to investigate gender, and the available studies are not in consensus. Since HLA-B27 molecules are major histocompatibility complex class I gene products which interact with T cells, in particular CD8+ T cells, it is conceivable that gender-related differences in immune response could play a role in the different manifestations of HLA-B27-associated uveitis. However, not all spondyloarthropathy patients with uveitis are positive for HLA-B27, so it seems likely that other factors are also important. Variable environmental exposures, either endogenous (i.e., sex hormone milieu) or exogenous (tendency towards exposure to infectious agents through lifestyle or different susceptibilities), might be expected to play roles as well. None of the studies collected data on pregnancy and its relationship to uveitis onset of recurrence in spondyloarthropathy patients. It is possible that hormonal fluctuations during and after the reproductive years play a role in gender differences as well. Again, these questions have not been investigated in the context of spondyloarthropathy and uveitis.

## 6. Conclusion

Few studies have investigated gender and spondyloarthropathy-associated uveitis. It is not surprising that males and females have different clinical characteristics, although we do not have much information to explain these differences or evaluate their importance. We do know that males are generally more likely to develop spondyloarthropathy and associated uveitis, probably at a younger age. Females who develop uveitis may have less classic HLA-B27-associated uveitis (i.e., insidious rather than abrupt onset) as well as atypical spondyloarthropathies (perhaps because “typical” was initially defined in male patients). Females also might be more likely to have frequent attacks of recurrent inflammation which require longer treatment. Fortunately, despite these clinical differences the rate of complications and vision loss seems to be unaffected. As we strive to develop a concept individualized medicine for uveitis patients, a better understanding of gender differences in clinical presentation, prognosis, and response to treatment may help us to provide better care and to prevent vision loss.

## Figures and Tables

**Table 1:** Summary of studies reporting data on gender in spondyloarthropathy and uveitis.

Study	Total number of patients	Female (%)	Type of spondyloarthropathy (if any)
Wakefield et al. 1984 [9]	40	21/40 (52.5%)	HLA-B27-negative AU
	41	9/41 (22%)	HLA-B27-associated AU*
		8/18 (44%)	Idiopathic HLA-B27-associated AU
		0/7 (0%)	AU + Reiters syndrome
		0/11 (0%)	AU + ankylosing spondylitis
		1/4 (25%)	AU + seronegative arthritis
		0/1 (0%)	AU + IBD

Rothova et al. 1992 [7]	73	21/73 (29%)	HLA-B27-associated AU

Linssen and Meenken 1995 [11]	35	18/35 (51%)	HLA-B27-negative AU
	119	43/119 (36%)	HLA-B27-associated AU*
		24/40 (60%)	Idiopathic HLA-B27-associated AU*
		16/44 (36%)	AU + ankylosing spondylitis*
		8/35 (23%)	AU + AS-related spondyloarthropathy*

Tay-Kearney et al. 1996 [12]	148	59^†^/148 (40%)	HLA-B27-associated AU*
		14/37 (38%)	AU + ankylosing spondylitis
		4/22 (18%)	AU + Reiters syndrome
		5/9 (55.5%)	AU + incomplete Reiters syndrome
		1/5 (20%)	AU + psoriatic arthritis and/or spondylitis
		2/3 (67%)	AU + IBD and/or spondylitis
		4/7 (57%)	AU + undifferentiated spondyloarthropathy

Lyons and Rosenbaum 1997 [19]	17	14/17 (82%)	Uveitis + IBD
	89^‡^	29/89 (33%)	Uveitis + spondyloarthropathy

Power et al. 1998 [3]	191	86/191 (45%)	HLA-B27-associated AU
		52/97 (54%)	AU without systemic disease
		17/51 (33%)	AU + ankylosing spondylitis
		5/26 (19%)	AU + Reiters syndrome
		4/5 (80%)	AU + psoriatic arthritis
		2/5 (40%)	AU + ulcerative colitis
		6/7 (86%)	AU + Crohn's disease

Paiva et al. 2000 [20]		5/16 (31%)	Uveitis + psoriatic arthritis (PsA)
		5/8 (62.5%)	Uveitis + PsA, and peripheral arthritis
		0/8 (0%)	Uveitis + PsA, peripheral and/or axial arthritis
	89^‡^	29/89 (33%)	Uveitis + spondyloarthropathy

Queiro et al. 2002 [21]	13	N.A.**	Uveitis + psoriatic arthritis

Monnet et al. 2004 [13]	175	76/175 (43%)	HLA-B27-associated AU
		22/39 (56%)	AU without spondyloarthropathy^†^
		54/136 (40%)	AU with spondyloarthropathy^†^
		42/98 (43%)	AU + ankylosing spondylitis or presumed AS
		8/21 (38%)	AU + undifferentiated spondyloarthropathy
		0/2 (0%)	AU + psoriatic arthritis
		2/6 (33%)	AU + reactive arthritis
		1/5 (20%)	AU + spondyloarthropathy and Behçet's disease
		1/4 (25%)	AU + inflammatory bowel disease

Braakenburg et al. 2008 [14]	177	81/177 (46%)	HLA-B27-associated AU
		15/39 (38%)	AU + HLA-B27-associated systemic disease
		10/30 (33%)	AU + ankylosing spondylitis

Chung et al. 2009 [15]	504	169/504 (33.5%)	HLA-B27-associated AU
		47/117 (40%)	AU without spondyloarthropathy
		46/214 (21.5%)	AU + ankylosing spondylitis
		69/150 (46%)	AU + undifferentiated spondyloarthropathy
		4/10 (40%)	AU + psoriatic arthritis
		3/11 (27%)	AU + reactive arthritis
		0/2 (0%)	AU + inflammatory bowel disease

Loh and Acharya 2010 [17]	99	43/99 (43%)	HLA-B27-associated AU
		18/44 (41%)	AU + HLA-B27-associated systemic disease

Agnani et al. 2010 [16]	207	91/207 (44%)	AU + HLA-B27 or axial spondyloarthritis
		55/126 (44%)	AU + axial spondyloarthritis

Zheng et al. 2012 [22]	240	71/240 (30%)	HLA-B27-associated AU (6 were HLA-B27 negative)
		19/108 (18%)	AU + ankylosing spondylitis
		9/16 (56%)	AU + undifferentiated spondyloarthropathy

AU: anterior uveitis, IBD: inflammatory bowel disease, AS: ankylosing spondylitis, PsA: psoriatic arthritis.

*All patients had HLA B27-associated AU plus one of the categories indicated.

^†^Data not available on systemic disease for all patients.

^‡^Same population of patients with uveitis and spondyloarthropathy used in each study.

**Number and percentage of females cannot be determined from publication.
